# Q-switched Nd:YAG Laser to Treat Nevomelanocytic Nevi

**DOI:** 10.4103/0974-2077.58521

**Published:** 2009

**Authors:** SG Parasramani, CM Oberai, KR Amonkar, S Naik

**Affiliations:** *Department of Dermatology, Venereology and Leprology, Lilavati Hospital, Mumbai, Maharashtra, India*

**Keywords:** Nevomelanocytic nevi, Q-switched Nd:YAG laser, Indian

## Abstract

**Background:**

Q-switched Nd:YAG laser, a melanin pigment-specific laser was used to treat melanocytic nevi in Indian patients.

**Objective:**

To assess the clinical response of nevi to Q-switched Nd:YAG laser of 1064 nm wavelength.

**Materials and Methods:**

Nineteen patients received an average of 2.57 treatments with Q-switched Nd:YAG laser of 1064 nm wavelength, repetition rate of 10 Hz, 10 nanoseconds pulse width, and spot size of 4 mm to 6 mm with a fluence ranging from 4 to 9.7 joules/cm^2^. The clinical end point of the laser treatment was brisk whitening. The response was assessed by using a global assessment score.

**Results:**

The clinical global assessment score showed excellent response in 8/19 patients and good response in 6/19 patients.

**Conclusion:**

The Q-switched Nd:YAG laser of 1064 nm wavelength resulted in significant improvement (lightening) of nevi.

## INTRODUCTION

Nevomelanocytic nevi (NMN), commonly called moles, are small, asymptomatic, circumscribed, pigmented macules, papules, or nodules, composed of groups of melanocytic nevus cells located in the epidermis, dermis, and rarely in the subcutaneous tissue. NMN can be congenital or acquired. Acquired NMN are classified as follows: a) Junctional melanocytic NMN, b) Compound melanocytic NMN, and c) Dermal melanocytic NMN.

Most NMN appear in early childhood and reach maximum size in young adulthood, while some may arise in adulthood. Later in adult life, these lesions involute and may disappear after the age of 60 years.[1] NMN have been treated by different methods such as surgical excision, electrocauterization, radio-frequency surgery, and carbon dioxide laser.

Leon Goldman was first to use the Ruby laser in 1960 for benign pigmented lesions. Later in 1983, Anderson and Parrish's theory of selective photothermolysis revolutionized laser therapy.[2] The chromophore targeted by the laser in this context is melanin. Melanosomes are 0.5 micrometers in size whereas a nevus cell is 10 micrometers in size.[3] As per the concept of thermal relaxation time (TRT), to minimize collateral thermal injury to the normal surrounding tissue, the pulses of light required to treat NMN must be very short. As the TRT of a melanosome is 0.25 microsecond and that of a nevus cell is 0.1 millisecond, lasers with pulse duration in the nanosecond range are used.[3] A laser of 1064 nm wavelength penetrates up to 2-3 mm, thus ensuring adequate dermal penetration.[4]

This study outlines our experience with the use of a 1064 nm Q-switched (QS) Nd:YAG laser in the treatment of nevomelanocytic nevi.

## MATERIALS AND METHODS

### Patient inclusion and exclusion criteria

Patients with at least five years history of pigmented nevi were included in the study. Additional criteria included uniformity in color, regular borders and the absence of any history of pruritus, bleeding or sudden increase in size, or surface textural change. All patients had no personal or family history of melanoma. Patients who were pregnant or lactating, had thyroid disorders, bleeding tendencies, keloidal tendency, *herpes simplex virus* infection, diabetes mellitus, hypertension and were on steroids, anticoagulants, or oral retinoids, were excluded.

Nineteen patients of skin types 5 and 6 were enrolled in the study.

### Laser treatment protocol

All patients were clinically examined in detail and asked to fill up a detailed questionnaire, which included the above mentioned inclusion and exclusion criteria. Informed consent and photographs of the patients were taken. Topical anesthesia (lidocaine and prilocaine combination) was applied and the area covered with micropore tape one hour before the laser treatment. Local infiltration anesthesia with 2% xylocaine was used in patients having large nevi. Patients' eyes were covered with protective shields while the operator used protective glasses. The area to be treated was disinfected and cooling was achieved by the use of a liquid nitrogen spray during the laser procedure. The following laser parameters were used: Q-switched Nd:YAG laser of 1064 nm wavelength, repetition rate of 10 Hz, pulse width of 10 nanoseconds, average spot sizes ranging from 3 to 6 mm were used. Fluence varied from 4.2 J/cm^2^ to 9.7 J/cm^2^ until immediate frosting was seen. NMN are small lesions and one has better control while treating them with a repetition rate of 2 and 5 Hz. This study used a repetition rate of 10 Hz, with one or two passes. After the laser treatment, an antibiotic dressing was given for 7-10 days along with analgesics, as needed, to control the pain until the wound had healed. Sunscreens were advised until the next session, which was after two months.

Photographic analysis of all patients was done in the same room and identical light conditions using a Nikon™ Digital Ds70 SLR camera. The patients were asked to do personal evaluation of the results. Melanin reflectance spectrometry and histopathological evaluation were not done.

### Global assessment score

Global assessment was done by comparing the photographs of treated nevi. Two blinded physicians independently assessed photographs and assigned a numeric score on a scale of 0 to 10 to each nevus (0 for No improvement to 10 for complete clearance).

## RESULTS

**Figure 1:**
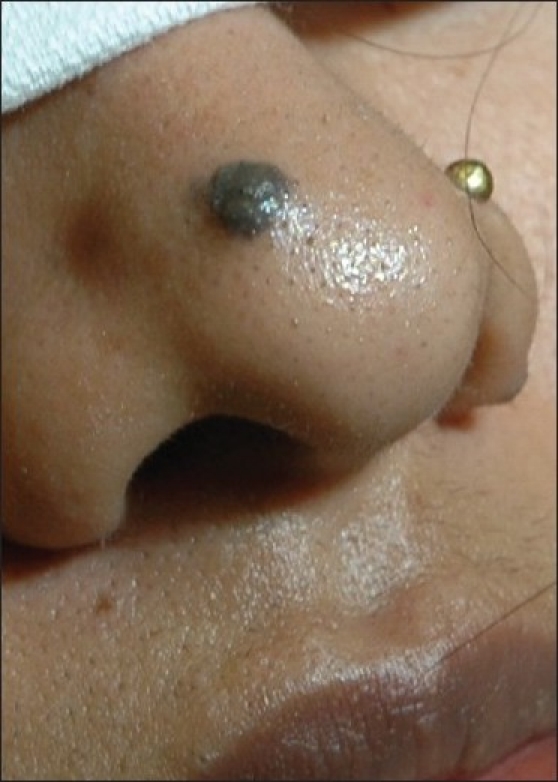
Compound NMN on tip of nose

**Figure 2:**
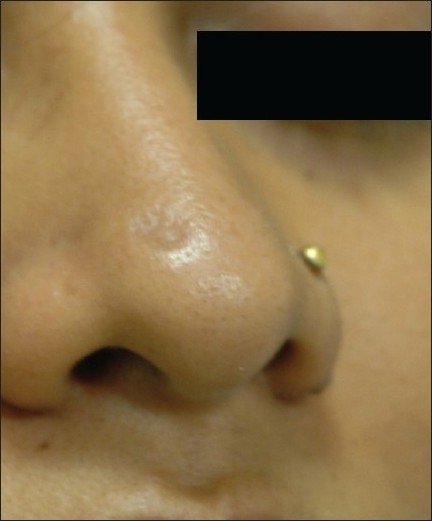
Compound NMN cleared completely after 4 sessions of laser with mild scarring (compare Figure 1)

**Figure 3:**
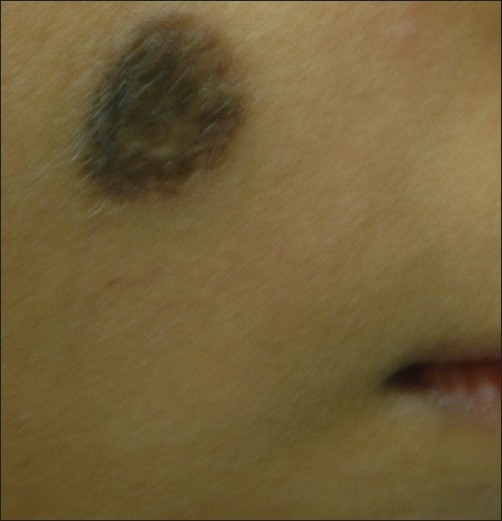
Pre-treatment photograph of congenital NMN

**Figure 4:**
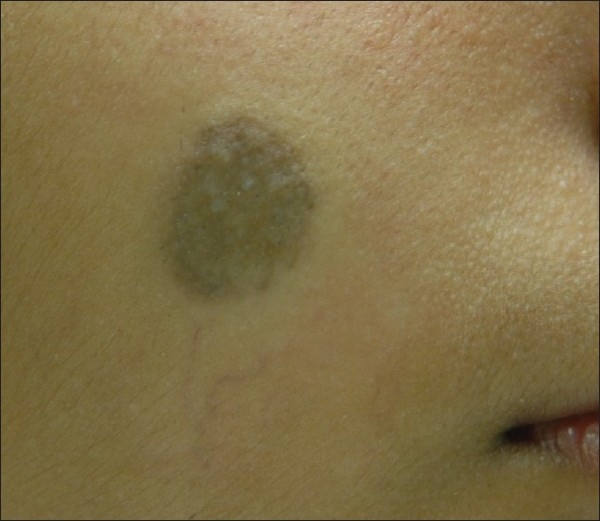
Improvement of congenital NMN achieved after 9 sessions of laser (compare Figure 3)

Nineteen patients were recruited in the study and were aged between 8-48 years (mean age of 25.27 years). Females predominated over males (M: F= 4.15) and most of the study subjects had acquired NMN (1, congenital NMN; 18, acquired NMN). Of the 18 with acquired NMN, six had junctional nevi, 11 had compound nevi, and one had a dermal nevus [Figures 1 and 2]. Two patients of the acquired NMN cohort dropped out of the study after one treatment. Of the remaining 16, 7 (7/16, 44%) showed over 80% improvement and another 8 (8/16, 50%) patients had 30-50% improvement, while one patient (1/16, 6%) had 10 % improvement [Table 1]. The patient with congenital NMN responded well (>90% clearance) after 10 sessions. There was no recurrence even after 8 months of followup [Figures 3 and 4].

**Table 1:** Percentage improvement achieved and number of sessions required in the acquired NMN cohort

% improvement	No of sessions	No of patients
100	1	1
90	1-10	6
80	4	1
50	2-3	4
40	1-3	2
30	1	2
Dropped out of study	-	2

The total number of treatments received by all 19 patients was 49, ranging from one to ten treatments (mean: 2.57).

One patient had complete clearance of nevi (100%) in just one treatment. Five patients had 90% clearance of nevi with treatment sessions ranging from 1-5 (mean: 2.8). One patient had 80% clearance in 4 treatments. Four patients had 50% clearance of nevi with treatment sessions ranging from 2-3 (mean: 2.25). Two patients had 40% clearance of nevi with treatment sessions ranging from 1-3 (mean: 2). Two patients had 30% clearance of nevi with only single treatment. One patient had 10% clearance after two treatments. Two patients had no clearance after one treatment and did not wish to undertake further treatments.

## DISCUSSION

Melanocytic nevi respond well to Q-switched (QS) lasers which produce ultra-short bursts of laser light to target melanin in the melanocytes. The laser produces an additional photoacoustic effect and high energy is delivered in a very short time leading to rapid thermal expansion of the target to produce the desired effect.[5] Three Q-switched lasers are available: Ruby 694 nm, Alexendrite 755 nm, and Nd:YAG laser 1064 nm. The last one is suitable for the treatment of darker skin types as it minimizes the risk of epidermal injury and pigmentary alteration.[6,7] This wavelength is weakly absorbed by epidermal melanin and has a deeper penetration into the dermis and is thus ideal to treat skin types 3-6.

On an average, 2.57 sessions were required to treat NMN with an interval of two months between two sessions. Lesions continued to clear, probably due to melanophages clearing the melanin from previously targeted melanocytes.[8] However, treatment of congenital NMN is controversial because of the very high number of pigment cells present, and lasers may not destroy all melanocytes particularly those in deeper layers.[9] There is an apprehension that these may harbor a potential for future malignant change and there is, therefore, much debate in this regard. It is also possible that removal of nevi, even partial, reduces the number of melanocytes and may decrease the potential of malignant change. In the past, melanocytic nevi have been inadvertently lased during treatments for other conditions, but malignant transformation has never been reported.[5]

The cosmetic benefit of QS laser irradiation surpasses the clinical effects of traditional treatments such as excision, as scarring is not seen with lasers. In a comparative study, Q-switched Alexandrite and Q-switched Nd:YAG lasers were studied in the treatment of benign NMN. Dramatic lightening of nevi was observed after three sessions with both treatments. Global assessment scores were better with QS Alexandrite laser than with QS Nd:YAG laser.[10]

Our study shows that QS Nd:YAG laser 1064 nm can be safely and effectively used in NMN. Larger comparative studies are needed with other lasers to establish a clear role of this type of laser treatment of nevi.
